# The ecological validity of MET was favourable in sitting implicit sequence learning consciousness by eyes closed and eyes open resting states fMRI

**DOI:** 10.1038/s41598-021-92616-y

**Published:** 2021-06-28

**Authors:** Jianxin Zhang, Xiangpeng Wang, Didi Zhang, Antao Chen, Dianzhi Liu

**Affiliations:** 1 School of Education, Jiangnan University, Wuxi, 214122 China; 2 School of Linguistic Sciences and Arts, Jiangsu Normal University, Xuzhou, 221009 China; 3 School of Education, Soochow University, Suzhou, 215123 China; 4 Key Laboratory of Cognition and Personality of Ministry of Education, Faculty of Psychology, Southwest University, Chongqing, 400715 China

**Keywords:** Consciousness, Human behaviour

## Abstract

The current study made participants sit to complete both the implicit sequence learning and the inclusion/exclusion tasks with the latter just after the former, and used eyes-closed and eyes-open resting states fMRI and their difference to test the ecological validity of the mutually exclusive theory (MET) in implicit-sequence-learning consciousness. (1) The behavioral and neuroimaging data did not support the process dissociation procedure, but did fit well with the MET. The correct inclusion-task response and the incorrect exclusion-task response were mutually exclusive with each other. The relevant brain areas of the two responses were either different or opposite in the eyes-closed and eyes-open resting-states and their difference. (2) ALFFs in eyes-closed and eyes-open resting-states and their difference were diversely related to the four MET knowledge in implicit sequence learning. The relevant brain areas of the four MET knowledge in the eyes-closed and eyes-open resting-state were the cerebral cortex responsible for vision, attention, cognitive control and consciousness, which could be called the upper consciousness network, and there were more relevant brain areas in the eyes-open resting-state than in the eye-closed resting-state.The relevant brain areas in ALFFs-difference were the subcortical nucleus responsible for sensory awareness, memory and implicit sequence learning, which could be called the lower consciousness network. ALFFs-difference could predict the four MET knowledge as a quantitative transition sensitivity index from internal feeling to external stimulus. (3) The relevant resting-state brain areas of the four MET knowledge were either different (for most brain areas, if some brain areas were related to one MET knowledge, they were not related to the other three MET knowledge) or opposite (for some brain areas, if some brain areas were positively related to one MET knowledge, they were negatively related to other MET knowledge). With the participants' control/consciousness level increasing from no-acquisition to controllable knowledge step by step, the positively relevant resting-state brain areas of the four MET knowledge changed from some consciousness network and the motor network, to some consciousness network and the implicit learning network, and then to some consciousness network; and the negatively relevant resting-state brain areas of the four MET knowledge changed from some consciousness network and visual perception network, to some consciousness network, then to some consciousness network and the motor network, and then to some consciousness network, the implicit learning network, and the motor network. In conclusion, the current study found the ecological validity of the MET was good in sitting posture and eyes-closed and eyes-open resting-states, ALFFs in eyes-closed and eyes-open resting-states and their difference could predict the four MET knowledge diversely, and the four MET knowledge had different or opposite relevant resting-state brain areas.

## Introduction

### The implicit-sequence-learning consciousness and its brain mechanism using the dichotomy

Implicit sequence learning is an important paradigm to reveal the mechanisms of consciousness^1–3^. There is a sequence rule in a certain dimension of stimulus, for example, location, but participants do not know the sequence rule and they are just asked to react stimulus location, therefore they do implicit sequence learning rather than explicit sequence learning. As the learning increases, different participants can generate knowledge at different consciousness levels for the sequence rule^4–6^, and a participant can also generate knowledge at different consciousness levels for different parts of the sequence rule^7^. One classical sequence rule is the second-order conditional sequence (SOC)^8^, which is a sequence such as 3-4-2-3-1-2-1-4-3-2-4-1, whereby each location is completely determined by the content of the previous two locations, i.e., when the previous two locations are 3-4, the next location must be 2; when the previous two locations are 4-2, the next location must be 3… Each three elements form a triplet, so there are 12 triplets.

Some neuroimaging studies explored implicit sequence learning. Primary somatosensory areas, premotor area, primary motor cortex, cerebellum and striatum were the brain areas activated in implicit sequence learning^9^. The cerebellum was the brain area of motor formatting optimization, motor control, and error correction; the primary motor cortex was the brain area of storing sequence knowledge; and the striatum was the brain area of stimulus–response connection learning and location prediction^10^.

Most neuroimaging studies used the dichotomy to explore sequence learning consciousness: Explicit sequence learning was defined as a conscious activity and implicit sequence learning as an unconscious activity. Therefore, the activated brain areas in explicit sequence learning or it minus the activated brain areas in implicit sequence learning was defined as consciousness brain areas, including the medial temporal lobe (including hippocampus)^11–14^, prefrontal lobe^15–19^ and insula^20^. Functional connections among attention and cognitive control networks increased after explicit sequence learning, while functional connections between the caudate nucleus and cingulate cortex increased after implicit sequence learning in the post-task resting-state compared to pre-task resting-state^21^. However, the dichotomy neither could distinguish learning processes from consciousness, nor recognized that implicit sequence learning could also produce many knowledge types, including consciousness. Compared with explicit sequence learning, implicit-sequence-learning consciousness goes through the process of consciousness generation from unconsciousness and upgrading. Thus, the dichotomy paradigm could not generate a continuous behavior/neuroimaging scale to quantify consciousness of implicit sequence learning^7^.

When unconscious knowledge became conscious in implicit sequence learning, functional connectivity between the right ventrolateral prefrontal cortex and ventral striatum existed^22–23^ . But these studies did not generate a continuous scale to quantify consciousness too.

### The PDP and its brain mechanism

The process dissociation procedure (PDP)^24–25^ provides a continuous scale to quantify consciousness in implicit sequence learning; accordingly, it is widely used^4–6,26–28^. It consists of free-generation tasks^29–30^ and trial-by-trial generation tasks^5,26,31^. Fu et al.^5,26^ set up the most concise trial-by-trial generation task: In the inclusion task, participants were presented with two trials of sequence fragments, and were asked to respond to the target quickly and accurately as in the learning phase. Then, four boxes with a question mark inside each were shown. Participants were asked to choose the location that conformed to the sequence rules. They could use conscious extraction (controlled response, shorthand for C) and unconscious familiarity (automatic response, shorthand for A) at the same time to make the correct inclusion response (C + A). In the exclusion task, participants needed to select a location that did not conform to the sequence rules. If participants wrongly select locations that did conform to the sequence rules, they constituted the incorrect exclusion-task response (A) which was only the automatic response driven by unconscious familiarity against conscious extraction. Therefore controlled response is that the correct inclusion-task response minus the incorrect exclusion-task response. The incorrect exclusion-task response (A) is contained within the correct inclusion-task response (C + A).

However, to date only a few studies have used the PDP with neuroimaging techniques in implicit sequence learning. Destrebecqz et al. (2005) found striatum activity was positively related to the incorrect exclusion-task response (automatic response), and anterior cingulate/central prefrontal cortex (ACC/MPFC) activity was positively related to correct exclusion-task response (controlled response). The automatic response occurred only in RSI (Response-Stimulus Interval) = 0 ms group, and there was functional connectivity between the ACC/MPFC and striatum in RSI = 250 ms group in which only controlled response occurred. The ACC/MPFC controlled activity of the striatum, and explicitly controlled its implicit components, which is the brain mechanism of consciousness^27^. However, the study did not identify brain areas associated with inclusion task. So it did not test the relationship between inclusion and exclusion tasks to test the PDP.

Huang et al. (2017) used task-state fMRI to find that in either the SOA (Stimulus Onset Asynchrony) = 850 ms group or the SOA = 1350 ms group, left medial frontal gyrus and left inferior parietal lobule were positively correlated to the correct inclusion-task response, but right inferior parietal lobule, left lingual gyrus and left inferior frontal gyrus were negatively correlated to the incorrect exclusion-task response^32^. In fact, these results showed that brain areas related to the correct inclusion-task response and the incorrect exclusion-task response were completely different or opposite to each other. However, the study did not point it out. It just used rather than test the PDP.

### The MET and its brain mechanism

Zhang et al.^33^ for the first time tested the PDP improved by Fu et al. (2010, 2013) by both behavior and neuroimaging with eyes-closed resting-state fMRI (Zhang et al. were also the authors of the current study). They pointed out that the PDP was not established. (1) In the inclusion task, participants must determine locations that conform to rules to choose them with both automatic and controlled responses. In the exclusion task, participants also must first incorporate both automatic and controlled responses to determine locations that conform to rules and then can avoid choosing them^5,26,34^. Therefore, cognitive activities by which participants determine locations that obey rules in exclusion tasks (the correct exclusion-task response) are identical to those in inclusion tasks (the correct inclusion-task response). That is, the incorrect exclusion-task response should completely differ from both the correct exclusion-task response and the correct inclusion-task response, which is completely contrary to the PDP hypothesis that the correct inclusion-task response contains the incorrect exclusion-task response. (2) The implicit cognitive control^35–36^ and the implicit sequence knowledge^5,26,34^ can be both automatic and controlled. That is, implicit knowledge is not only characterized by automatic response. But the PDP hypothesizes only one automatic implicit knowledge type, which is inconsistent with those studies.

Therefore they proposed the mutually exclusive theory (MET) that the correct inclusion-task response and the incorrect exclusion-task response are either independent or in opposition to each other. They are likely to be either not correlated or negatively correlated, and the relevant brain areas of them should be either not overlap or in opposition to each other. The correct inclusion-task response is equivalent to the correct exclusion-task response, which is equal to C + A_1_, and the incorrect exclusion-task response is equal to A_2_. On the contrary, because the PDP states that the correct inclusion-task response (C + A) contains the incorrect exclusion-task response (A), the former should not be less than the latter, they are less likely to be negatively correlated, and the relevant brain areas of them should be partially overlap. In their study, the behavioral data and neuroimaging data were perfectly consistent with the MET, but were completely contrary to the PDP. So they used the MET to categorize the 12 kinds of triplets as four MET knowledge, namely non-acquisition of knowledge, uncontrollable knowledge, half-controllable knowledge, and controllable knowledge (please see “Behavioral and neuroimaging results of the MET” in the current study for details). The participants’ control of the four MET knowledge gradually improved from non-acquisition to controllable knowledge. Correspondingly, the positively-relevant resting-state brain areas of the four MET knowledge gradually changed from the sensory and motor network to the somatic sensorimotor network, and then to the implicit learning network, and then to the consciousness network. The negatively-relevant resting-state brain areas of the four MET knowledge gradually changed from the consciousness network to the sensory and motor network.

### Resting-state fMRI suitable for studying implicit-sequence-learning consciousness

Resting-state fMRI investigates spontaneous activity or functional connections of the brain at rest, which is well suited to studying consciousness, The resting-state rationales are as follows^37–40^: If a cognitive task is related to some resting-state brain areas, these brain areas can be considered to be responsible for the cognitive task. If the relevant resting-state brain areas of two cognitive tasks differ, the brain mechanisms of these cognitive tasks are different. The relevant resting-state brain areas of a cognitive task are usually proved to be activated by task-state fMRI when the cognitive task is carried out^21–23,27,32–33^, which is also holds true for the current study, see results and discussion for details. Therefore brain spontaneous activity in resting-state is a stable indicator of individual cognitive characteristics^38^. A classical index is the amplitude of Low Frequency Fluctuations (ALFFs, 0.01–0.1 HZ), including most of the psychological cognitive process. The higher and lower frequencies are background noise. There were eyes-closed and eyes-open resting-states. Yan et al.^41^ found that although the connectivity patterns of the DMN (the defaultmode network) were visually similar across eyes-closed (EC), eyes-open with no fixation (EO) and eyes-open with a fixation (EO-F), there was significantly higher functional connectivity and ALFF in both the EO and the EO-F conditions as compared to the EC condition, which suggested that the participants might have more non-specific or non-goal-directed visual information gathering and evaluation, and mind wandering or daydreaming during the eyes-open resting state. Nakano, etc. (2012, 2015) found that internal feeling and self-consciousness were focused on in eyes-closed resting state,, while external stimulus processing was focused on in eyes-open resting state. When participants switched from eyes-closed to eyes-open, transition occurred from internal feeling and self-consciousness to external stimulus processing^42–43^. However, no study has taken their difference, for example, ALFFs in eyes-open resting-state minus ALFFs in eyes-closed resting-state, as a quantitative transition sensitivity index from internal feeling to external stimulus or investigated the relationship between it and other cognition or behavior, although the above literatures investigated its own psychological significance^41–43^.

Sleep and disturbed consciousness resting-state studies found that there is a global consciousness network named the "rich club", including the dorsolateral prefrontal cortex, inferior parietal lobe, middle temporal lobe, precuneus, insula, thalamus, and brainstem^44–47^. However, there exists only a few resting-state researches in implicit-sequence-learning consciousness. Sami et al.^21^ used pre- and post-task resting-states changes to study functional connection differences between implicit and explicit sequence learning. Because the study used the dichotomy, it did not assess consciousness in implicit sequence learning and could not separate implicit sequence learning from consciousness (Please see “The implicit-sequence-learning consciousness and its brain mechanism using the dichotomy” for details). Zhang et al.^33^ tested the PDP and explored relevant resting-state brain areas of the MET knowledge with eyes-closed resting-state fMRI (Please see “The MET and its brain mechanism” for details), but they did not consider the resting-state types and the ecological validity of the MET.

### The ecological validity of the MET tested in the current study

Although Zhang et al.^33^ successfully tested the PDP and presented a more logical and data-friendly MET theory to quantify the MET knowledge in implicit sequence learning by both behavior and neuroimaging with eyes-closed resting-state fMRI, there were problems with the ecological validity of the MET. (1) Participants completed implicit sequence learning while lying down and receiving task-state scanning. But the implicit sequence learning in previous studies and real life happened when the upper body was upright. Is the MET suitable for implicit sequence learning when the upper body is upright? (2) After the learning phase, participants received 8 min post-task resting-state scanning before the inclusion/exclusion tasks. But participants did the inclusion/exclusion tasks just after the implicit sequence learning in previous studies. Is the MET suitable for the inclusion/exclusion tasks just after the implicit sequence learning? (3) Participants sat in front of a computer to do the inclusion/exclusion tasks, which was not consistent with lying-down body posture in implicit sequence learning. But participants did the inclusion/exclusion tasks with the same sitting body posture as the implicit sequence learning in most previous studies. Is the MET suitable for sitting posture in both the implicit sequence learning and the inclusion/exclusion tasks? (4) ALFFs in eyes-closed resting-state were related to the MET knowledge in implicit sequence learning. But participants did both the implicit sequence learning and the inclusion/exclusion tasks with eyes-open. In implicit sequence learning and its consciousness, vision plays an important role. Eyes-open resting-state contains visual information gathering and evaluation^41–43^. Eyes-open resting-state minus ALFFs in eyes-closed resting-state contains visual consciousness and the transition from internal feeling and self-consciousness to external stimulus processing^41–43^. Is the MET suitable for both eyes-open resting-state and eyes-closed resting-state and their transition(difference)?

In conclusion, the ecological validity of the MET needs to be tested. Therefore, the current study made participants sit to do both the implicit sequence learning and the inclusion/exclusion task with the latter just after the former, and used eyes-closed and eyes-open resting-states and their difference as a quantitative transition sensitivity index from internal feeling to external stimulus to test the ecological validity of the MET in implicit sequence learning. These settings were closer to implicit sequence learning in previous researches and real life. If the MET stays true, it indicates that its ecological validity is good. At the same time, the current study could reveal a rich resting state brain mechanism of the MET knowledge.

## Materials and methods

### Participants

Sixty five college students participated in the experiments. All participants were right-handed, with normal or corrected-to-normal vision, normal color perception, normal physical and mental health, were not taking psychotropic drugs, and had never previously participated in any implicit-learning experiment. All participants met the criteria for functional magnetic resonance imaging (fMRI) scanning, namely that they had no metal implants, were not claustrophobic, and had a head size compatible with the head coil. There were 32 males and 33 females, with age *M* ± *SD* = 21.71 ± 2.58. Participants volunteered to take part in the experiment. Each participant completed an informed consent form before the experiment, and got paid after the experiments. The experiments were in accordance with the ethical guidelines of the Declaration of Helsinki, and were approved by the Scientific Review Committee of Faculty of Psychology, Southwest University, China.

### Materials

Implicit sequence learning materials were four blue circles (4.6 cm in diameter), which were arranged horizontally. The centers of adjacent circles were 6.9 cm apart, and the left two circles were symmetrical with the right two circles relative to the center of the screen. Only the target circle was filled in blue; the other circles were outlined in blue, with unfilled centers. The target-circle location order followed the SOC sequence rule: 3-4-2-3-1-2-1-4-3-2-4-1^5,8,26,33^, in which each target location was determined by two prior target locations, and three target locations formed a smallest-rules unit, namely a triplet.

### Design and procedure

**Figure 1:**
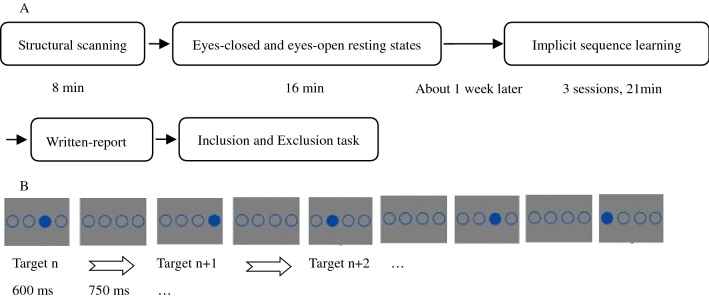
A, flow chart of all experiments; B, stimulus presentation of implicit sequence learning.

The current study used a single variable (resting-state: eyes-closed vs. eyes-open) within-subjects design. The participants were required to complete all tasks strictly and carefully in accordance with the instructions, otherwise they would not get paid. The experimental procedure is shown in Fig. 1.

The participants accepted structural scanning, eyes-closed and eyes-open resting states scanning in the fMRI scanner. About one week later, they were asked to do implicit sequence learning tasks. They sat in front of a computer, with their eyes 70 cm from the center of the screen. A custom experimental program ran under E-prime 2.0 on a PC (Lenovo LX-GJ556D), with a 17-inch color display (resolution 1024 × 768, refresh rate 60 Hz). They were asked to put the middle finger and index finger of their left hand on key D and F, and the index finger and middle finger of their right hand on key J and K, corresponding to the horizontal positions of the stimulus circles. In the implicit sequence learning phase, instructions stated that the experiment measured response speed and accuracy of pressing a target-location button. The task was to immediately press the button corresponding to the position of the solid circle as quickly and accurately as possible. In the practice stage, there were 24 trials that obeyed the SOC rule. The reason for not using a random sequence was to avoid such a random sequence from influencing implicit sequence learning as a novel stimulus. Then, there were 15 blocks of implicit sequence learning. Each block was composed of 48 trials with a different target-location in the first trial to avoid participants easily noticing the sequence rules. The SOC rule cycled 4 times in each block. Before the start of each block, a fixation cross was presented for 13.2 s as a baseline. Five blocks formed a session. Before each session a fixation cross was shown for 7.5 s. There was a 40 s rest period between each two sessions. A solid circle and three unfilled circles were also shown 600 ms, followed by four unfilled circles for 750 ms; participants were required to make their selection within SOA = 1350 ms.

After the learning phase, the consciousness-assessment stage included a written-report task, an inclusion task, and an exclusion task. In the written-report task, participants were asked to write down on a piece of paper all thoughts they had while they were taking part in experiment. The inclusion/exclusion task was the same as that in Zhang et al.^33^, Fu et al.^26^ and Fu et al.^5^. In the third element of each triplet, when four boxes with a question mark inside each were shown, participants could not choose the same location as the second element. Therefore, there were only three locations they could choose, and the chance performance level was one-third. The inclusion/exclusion task consisted of 72 triplets of the sequence, such that the full set of 12 kinds of triplets of the SOC rule were repeated 6 times. The ABBA design was used for the inclusion/exclusion task to balance the order effect.

### Resting-state data collection and analysis

All the participants first received the structural scan, then half received the eyes-closed and eyes-open resting-state scans, and half received the eyes-open and eyes-closed resting-state scans. The fMRI data were collected using a Siemens 3.0 T magnetic resonance imaging scanner and an 8-channel phased front head coil. Eyes-closed and eyes-open resting-state imaging used gradient echo (GRE) single-excitation echo-planar imaging (EPI). Scan parameters were as follows: TR = 2000 ms, TE = 30 ms, FA = 90°, FOV = 220 × 220 mm^2^, matrix size = 64 × 64 mm^2^, depth = 3 mm, planar resolution = 3.13 × 3.13 mm^2^, interval scanning, 33 layers, layer spacing = 0.6 mm, total 240 layers. Structural imaging used a 3D TlWI (MP-RAGE) sequence with sagittal scans. Scan parameters were the following: TR = 2600 ms, TE = 3.02 ms, FA = 8°, no interval, FOV = 256 × 256 mm^2^, matrix size = 256 × 256 mm^2^, total 176 layers.

Pretreatment and analysis of resting-state data used DPARSF 3.0^48^ Advanced Edition Calculate in Original Space (Warp by DARTEL), following standard procedures: First, conversion of raw DICOM-format data to NIFTI format. To allow for signal stabilization of the image, the first 10 TR images were removed, after which time layer correction (slice timing) and head movement correction (realign) were conducted. If head movement greater than 2 mm occurred during resting-state, the data were deleted. Second, the new segment and DARTEL was used to split the structural T1 data without standardization, and register the T1 split data directly to the resting-state functional images. Before registration of structural and functional data, the AC-PC line of each participant’s T1 image and the resting-state function was registered, and then automatic registration was applied. Thus, the resting-state analysis took place in the original T1 space. Third, head motion (adopting Friston 24), linear drift, white matter and cerebrospinal fluid were adjusted via regression. Fourth, low frequency fluctuations ALFFs (filter range: 0.01 to 0.1 Hz) were calculated. Fifth, the resting-state function was registered to the standard MNI space (normalization), using a 3 × 3 × 3 mm^3^ voxel size, with 4 × 4 × 4 mm^3^ full width at half maximum (FWHM) smoothing.

REST1.8^49^ was first used to extract ALFFs in eyes-closed and eyes-open resting-states in 116 Anatomical Automatic Labeling (AAL) brain areas. Second, SPSS19.0 was used to implement correlation analyses between ALFFs in eyes-closed and eyes-open resting-states and their ALFFs-difference (ALFFs in eyes-open resting-state minus ALFFs in eyes-closed resting-state) in 116 AAL brain areas and the PDP. Finally, SPSS19.0 was used to generate Pearson correlation analyses between ALFFs in eyes-closed and eyes-open resting-states and their ALFFs-difference in 116 AAL brain areas and the MET knowledge. Since the original ALFF of each AAL brain area (the average ALFF of its all voxels) was extracted^33,50–52^, multiple comparison correction was not required for the correlation analyses above. The principle of multiple comparison correction is as follows: Suppose that a nucleus contains 10 voxels, in order to ensure the *p* value of correlation coefficient between the nucleus and a certain cognitive index is less than 0.05, the *p* value of correlation coefficient between the cognitive index and each voxel of the 10 voxels should be less than 0.005. The making-mistake probability of correlation coefficient between the cognitive index and the nucleus of 10 voxels is less than 0.005 × 10 = 0.05^53^. Therefore, if we get each qualified voxel to obtain the relevant nucleus of the cognitive index, the multiple comparison correction must be carried out. However, the current study did not use this voxel-correlation method; it directly extracted the original ALFF for a certain AAL brain area (the average ALFF of its all voxels), and then calculated correlation coefficient between the original ALFF of this AAL brain area and a certain cognitive index. There was only one correlation calculation for this AAL brain area, so it was unnecessary and impossible to carry out any multiple comparison correction. This logical and computational approach was appreciated by some researches^33,50–52^.

It should be noted that the AAL template depends on the anatomical structure, and many brain regions have large morphological structure, which will average the results within the regions and easily conceal some very significant differences with a small brain area. Therefore, a more refined approach is the voxel-correlation method to correlate each voxel with the cognitive index, and then carry out multiple comparison correction to obtain the relevant brain nucleus of the cognitive index. However, the current study need compare the relevant resting-state brain areas of the four MET knowledge types in eyes-closed and eyes-open resting-states and their difference. If the voxel-correlation method is used, the relevant resting-state brain nucleus of each MET knowledge will be too complex and trivial to compare. Of course, collinearity analysis can reduce these defects to some extent. But the differences among the four MET knowledge and between the two resting-states can be revealed clearly and concisely by using AAL template: If an AAL area is related to neither of two MET knowledge, it can be presumed that this AAL area is not responsible for any of the two MET knowledge; If an AAL area is related to one MET knowledge, but is not related to another MET knowledge, it can be presumed that they have different cognitive component; if an AAL area is positively related to one MET knowledge, but is negatively related to another MET knowledge, it can be presumed that they have competing cognitive component; if an AAL area is positively/negatively related to two MET knowledge, it can be presumed that they partly have the same cognitive component. In addition, although the AAL template will average the results within the regions and easily conceal some very significant differences with a small brain area, this average is just enough to reduce random error and obtain large-scale correlation through coarse graining, which is suitable for consciousness governed by large-scale brain areas^40–43^. Therefore, the current study used the AAL template.

Although Curtin and Schulz^54^ pointed out multiple correlations need multiple correction, for example, Bonferroni's correction, we thought multiple correction is too strict to reveal real correlations. Assuming that we used multiple comparisons, since each MET knowledge was made Pearson correlation analysis with 116 AAL brain areas, the *P* value of each correlation coefficient should be less than 0.05/116 = 0.00043, in order to ensure that the α mistake probability of all correlations was less than 0.05. That was too strict to leave any correlation, even using less conservative false-discovery-rate multiple comparisons^55^. Therefore Zhang et al.^56^ did not make multiple correction for Pearson correlation among multiple AAL brain areas, but used a threshold of α = 0.05 for testing all graph characteristics in the functional connectivity network construction. Zhang et al.^33^ also used the same threshold of α = 0.05 for testing all correlations between each MET knowledge and 116 AAL brain areas. Logically analysis, in the current study, although each MET knowledge was made correlation analysis with 116 AAL brain areas, the relevant AAL brain areas need not be bundled to form a larger relevant brain areas as voxel-wise, so each correlation analysis was a separate event, and each correlation analysis had a separate α mistake probability and did not interact with each other. There was no reason to make multiple correction.

## Results

SPSS 19.0 was used for statistics. All participants had an accuracy of no less than 90% in the implicit sequence learning phase^6,33,57^, indicating that they completed implicit sequence learning seriously. Because one participant’ learning extent was at chance, seven participants’ head movement were greater than 2 mm in eyes-closed resting-state, and three participants in eyes-open resting-state, there were 64 valid participants in implicit sequence learning, 57 valid participants in eyes-closed resting-state, and 61 valid participants in eyes-open resting-state. To investigate consciousness in pure implicit sequence learning, in the learning phase none of the novel stimuli presented, such as improbable sequences or transfer blocks^19,58–59^ Therefore, there was no reaction-time difference between probable and improbable sequences to be used as learning extent. Because of practice and fatigue effects, reaction-time difference between block 1 and 15 could not serve as learning extent. Instead, we used indexes in the generation tasks to estimate learning^32–33^.

### Behavioral and neuroimaging results of the PDP

#### The PDP analysis for generation tasks

Because both the correct inclusion-task response and the incorrect exclusion-task response are measures of the learning degree, the greater of them was used as learning extent (see Table 1). If a participant’s correct inclusion-task response was no less than his/her incorrect exclusion-task response, the former was used as his/her learning extent; otherwise, the latter was used.

**Table 1:** Indexes of generation tasks (*M* ± *SD*).

	*M* ± *SD* (*n* = 64)	*t*	Cohen's *d*
the correct inclusion-task response	34.45 ± 7.57	12.11***	1.38
the incorrect exclusion-task response	25.42 ± 10.12	1.12	–
Learning extent	37.00 ± 5.88	17.69***	2.21

Chance performance for the inclusion/exclusion tasks was one-third. There were 72 triplets in the inclusion/exclusion tasks, so that the chance performance for each task was 24 correct/incorrect response. Therefore single-sample *t*-test was used to determine which index was greater than 24. Learning extent was greater than chance, which suggests that implicit sequence learning occurred. Each participant’s learning extent was also greater than 24, which suggests that each participant produced implicit sequence learning. The correct inclusion-task response was greater than chance, but the incorrect exclusion-task response was not greater than chance, which indicates that there was only correct inclusion-task response but no incorrect exclusion-task response in the group as a whole. The incorrect exclusion-task response occurred less frequently than the correct inclusion-task response, which was consistent with both the PDP and the MET.

Further, Pearson correlation was made between the correct inclusion-task response and the incorrect exclusion-task response. They were negatively associated, *r* (65) = -0.58, *p* < 0.001. This indicates that the two responses were in opposition to each other. The PDP states that the correct inclusion-task response contain the incorrect exclusion-task response^5,24–25,26^; that is, they can be either positively(most likely), negatively (less likely) or not associated. So the PDP could explain the negative correlation result, but the result could not prove the PDP. The MET proposed by Zhang et al.^33^ was better suited for this negative correlation result, which states that the correct inclusion-task response (C + A_1_) and the incorrect exclusion-task response (A_2_ are mutually exclusive; that is, they can only be either negatively associated (most likely or not correlated (because of random error, but they can never be positively associated.

#### Resting-state brain activity related to the PDP

Although the incorrect exclusion-task response was at chance in the group as a whole, there might exist individual difference. Therefore, the group was half-splited into high and low subgroups on the incorrect exclusion-task response^33^. The high and low subgroups differed significantly (independent-groups *t*-test, *p* < 0.001), whereby the high subgroup performed better than chance (*p* < 0.001). This indicates that individual difference was present: Some participants gained sequence rule knowledge with higher consciousness level, so they could control their knowledge to avoid producing the incorrect exclusion-task response; but some participants gained sequence rule knowledge with lower consciousness level, so they could not control their knowledge and produced the incorrect exclusion-task response. Therefore it was meaningful to carry out correlation analyses between the incorrect exclusion-task response and ALFFs in resting states to explore which brain areas were responsible for the individual difference. In some cases, when the whole group of participants does not show a certain feature, it does not mean that every participant in the group does not show this feature. Therefore, it is necessary to discuss which participants in the group show this feature and which participants do not show this feature, and what the reason is. This proof is so fundamental that it is often implied and concealed, which was proposed by our published paper last year^33^. When the ceiling effect or floor effect occurs, the above method will find no difference between the high and low subgroups. If there is no difference in the incorrect exclusion-task response between the high and low subgroups, or if the incorrect exclusion-task response in the high subgroup is still no higher than chance, it means that none of the participants have any incorrect exclusion-task response, and it makes no sense to associate the "non-existent" incorrect exclusion-task response of these participants with their ALFFs in resting states. The subgroups were only made to test the individual differences. Therefore, they were not considered any more in the following analysis.

Some AAL areas were related to the correct inclusion-task response, but they were not related to the incorrect exclusion-task response, and vice versa; Caudate_L in ALFFs-difference was negatively related to the correct inclusion-task response, but was positively related to the incorrect exclusion-task response. It indicates that the two responses types were either independent or competing (see Table 2). These results were inconsistent with the PDP, but were consistent with the MET. Therefore, the MET was detected in the following.

**Table 2:** Correlations between ALFFs/ALFFs-difference with the PDP.

	AAL brain area	ALFFs	*r*_in_	*r*_ex_
Eyes-closed	Frontal_Sup_Medial_R	0.93 ± 0.06	− 0.27*	
Eyes-closed	Fusiform_R	0.87 ± 0.03		− 0.30*
Eyes-closed	SupraMarginal_L	0.89 ± 0.04	0.38**	
Eyes-closed	Angular_L	1.03 ± 0.07	0.37**	
Eyes-closed	Cerebelum_Crus2_L	0.66 ± 0.19	− 0.27*	
Eyes-closed	Vermis_7	0.81 ± 0.07	− 0.26*	
Eyes-closed	Vermis_9	1.11 ± 0.26	0.26*	
Eyes-open	SupraMarginal_L	0.89 ± 0.03	0.27*	
Eyes-open	Angular_L	1.05 ± 0.06	0.41**	
Eyes-open	Thalamus_L	1.00 ± 0.08	− 0.31*	
Eyes-open	Thalamus_R	0.99 ± 0.07	− 0.34**	
Eyes-open	Cerebelum_Crus1_R	0.97 ± 0.10		− 0.28*
ALFFs-difference	Frontal_Sup_L	0.01 ± 0.04	0.27*	
ALFFs-difference	Frontal_Mid_L	0.01 ± 0.04	0.27*	
ALFFs-difference	Frontal_Mid_R	0.01 ± 0.03	0.29*	
ALFFs-difference	Insula_R	0.01 ± 0.02		0.36**
ALFFs-difference	Cingulum_Ant_L	0.01 ± 0.04	0.31*	
ALFFs-difference	Hippocampus_L	0.01 ± 0.03	− 0.35**	
ALFFs-difference	ParaHippocampal_L	0.01 ± 0.06		0.28*
ALFFs-difference	Caudate_L	0.01 ± 0.04	− 0.35**	0.33*
ALFFs-difference	Cerebelum_4_5_R	− 0.02 ± 0.05	− 0.28*	

*r*_in_ is the correlation between ALFFs/ALFFs-difference and the correct inclusion-task response; *r*_ex_ is the correlation between ALFFs/ALFFs-difference and the incorrect exclusion-task response. *p < 0.05, **p < 0.01, ***p < 0.001. The same below.

### Behavioral and neuroimaging results of the MET

#### The MET analysis for generation tasks

Each of the 12 kinds of triplets in the SOC repeated 6 times in the inclusion/exclusion task. So for a participant, each kind of triplet had a correct inclusion-task response and a incorrect exclusion-task response, whose chance performance was one-third, namely 2 times. The MET was used to categorize the 12 kinds of triplets as four MET knowledge, namely non-acquisition of knowledge, uncontrollable knowledge, half-controllable knowledge, and controllable knowledge by comparing the relationship between the two responses for each triplet (see Table 3)^33^. The participants' control and consciousness level was gradually increasing from no-acquisition to uncontrollable knowledge, then to half-controllable knowledge, and then to controllable knowledge.

**Table 3:** The four MET knowledge.

Types of knowledge	the correct inclusion-task response	the incorrect exclusion-task response	*M* ± *SD* (*n* = 64)
no-acquisition	≤ 2 (at chance)	≤ 2 (at chance)	2.30 ± 1.43
uncontrollable knowledge	≤ 2 (at chance)	≥ 3 (greater than chance)	3.06 ± 1.94
half-controllable knowledge	≥ 3 (greater than chance)	≥ 3 (greater than chance)	1.44 ± 1.53
controllable knowledge	≥ 3 (greater than chance)	≤ 2 (at chance)	5.20 ± 2.09

A repeated-measures analysis of variance was carried out for the four types of knowledge. Sphericity was significant (*p* < 0.001), then Greenhouse correction was made and there was a significant main effect of knowledge type, *F* (2.49, 61) = 39.82, *p* < 0.001, η_p_^2^ = 0.387. After Bonferroni correction for multiple comparisons, there was significantly less half-controllable knowledge than the other three knowledge (*p*s < 0.01), and that there was significantly more controllable knowledge than the other three knowledge (*p*s < 0.001).

#### Resting-state brain activity related to the four MET knowledge

**Figure 2:**
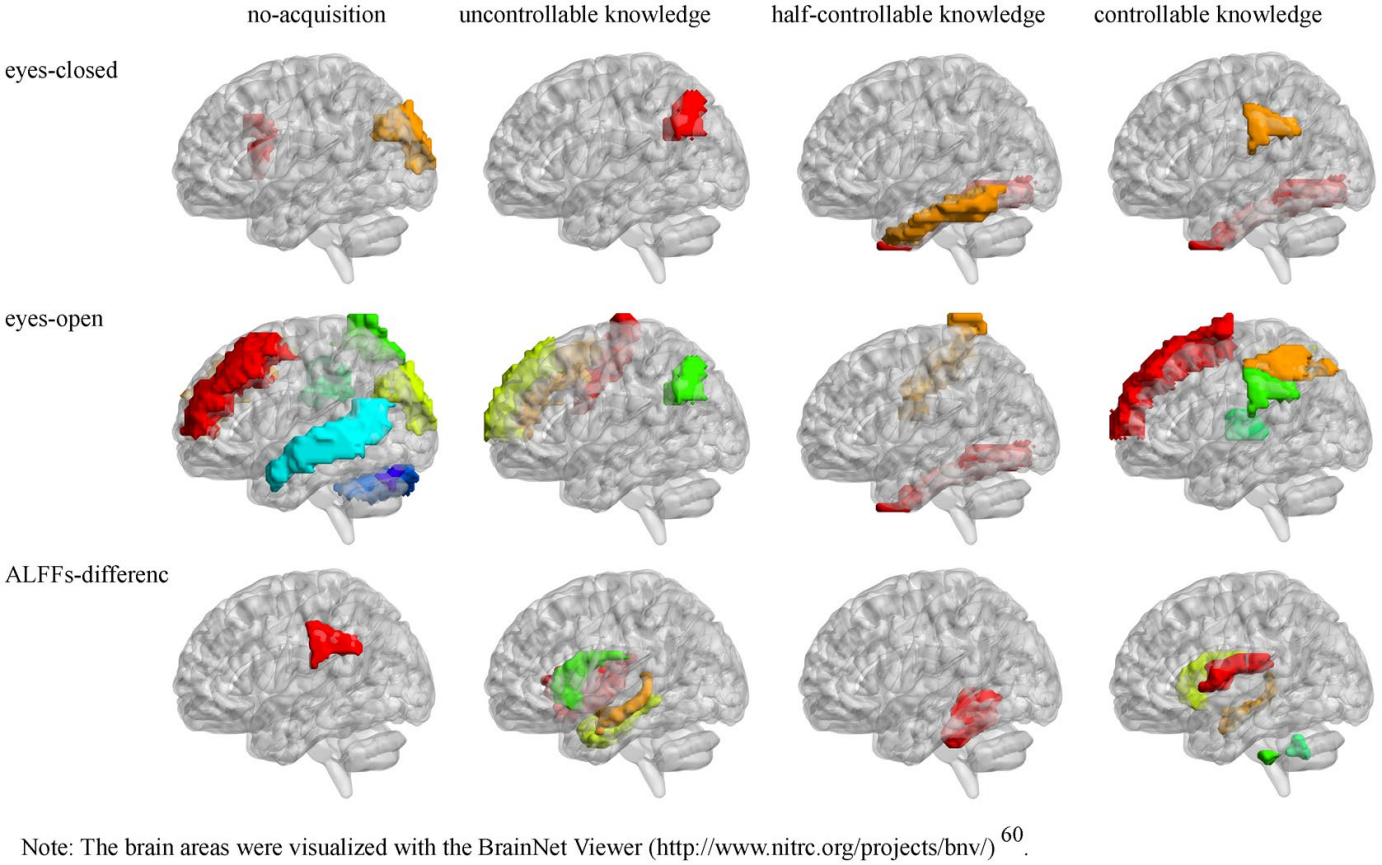
AAL brain areas whose ALFFs/ALFFs-difference were related to the four MET knowledge. *Note*: The brain areas were visualized with the BrainNet Viewer (http://www.nitrc.org/projects/bnv/) ^60^.

Resting-state brain activity was related to the four MET knowledge (see Table 4 and Fig. 2).

**Table 4:** Correlations between ALFFs/ALFFs-difference with the four MET knowledge.

	AAL brain area	ALFFs	*r*_no_	*r*_un_	*r*_half_	*r*_con_
Eyes-closed	Frontal_Inf_Oper_R	0.88 ± 0.04	0.28*			
Eyes-closed	Occipital_Sup_L	0.92 ± 0.08	− 0.27*			
Eyes-closed	Fusiform_R	0.87 ± 0.03			− 0.30*	0.33*
Eyes-closed	SupraMarginal_L	0.89 ± 0.04				0.40**
Eyes-closed	Angular_L	1.03 ± 0.07		− 0.31*		
Eyes-closed	Temporal_Inf_L	0.81 ± 0.04			− 0.29*	
Eyes-open	Precentral_R	0.85 ± 0.05		0.27*		
Eyes-open	Frontal_Sup_L	0.87 ± 0.05				0.26*
Eyes-open	Frontal_Mid_L	0.88 ± 0.05	− 0.26*			
Eyes-open	Frontal_Mid_R	0.88 ± 0.04	− 0.26*	0.29*		
Eyes-open	Frontal_Sup_Medial_R	0.94 ± 0.06		0.33*		
Eyes-open	Occipital_Sup_L	0.92 ± 0.06	− 0.27*			
Eyes-open	Fusiform_R	0.87 ± 0.03			− 0.26*	
Eyes-open	Postcentral_R	0.84 ± 0.05			− 0.26*	
Eyes-open	Parietal_Sup_R	0.94 ± 0.07	− 0.26*			
Eyes-open	Parietal_Inf_L	0.99 ± 0.05				0.26*
Eyes-open	Parietal_Inf_R	1.06 ± 0.06				0.26*
Eyes-open	Cerebelum_Crus1_R	0.97 ± 0.10	0.30*			
Eyes-open	Vermis_7	0.82 ± 0.06	0.28*			
ALFFs-difference	Rolandic_Oper_L	0 ± 0.02				− 0.30*
ALFFs-difference	Insula_R	0.01 ± 0.02		0.30*		
ALFFs-difference	Hippocampus_L	0.01 ± 0.03		0.29*		
ALFFs-difference	Hippocampus_R	0.01 ± 0.02				− 0.28*
ALFFs-difference	ParaHippocampal_L	0.01 ± 0.06		0.26*		
ALFFs-difference	SupraMarginal_L	0 ± 0.03	0.31*			
ALFFs-difference	Caudate_L	0.01 ± 0.04		0.35**		− 0.31*
ALFFs-difference	Caudate_R	0.01 ± 0.03		0.28*		
ALFFs-difference	Cerebelum_4_5_L	− 0.01 ± 0.05			− 0.28*	
ALFFs-difference	Cerebelum_10_L	0.04 ± 0.21				− 0.27*
ALFFs-difference	Vermis_9	− 0.01 ± 0.09				− 0.28*

*r*_no_ is the correlation between ALFFs/ALFFs-difference and no-acquisition; *r*_un_ is the correlation between ALFFs/ALFFs-difference and uncontrollable knowledge; *r*_half_ is the correlation between ALFFs/ALFFs-difference and half-controllable knowledge; *r*_con_ is the correlation between ALFFs/ALFFs-difference and controllable knowledge.

No-acquisition: (1) In the eyes-closed resting state, some frontal lobe (Frontal_Inf_Oper_R) was positively related to no-acquisition; some occipital lobe (Occipital_Sup_L) was negatively related to no-acquisition. (2) In the eyes-open resting state, some cerebelum and vermis (Cerebelum_Crus1_R, Vermis_7) were positively related to no-acquisition; some frontal lobes (Frontal_Mid_L, Frontal_Mid_R), occipital lobe (Occipital_Sup_L) and parietal lobe (Parietal_Sup_R) were negatively related to no-acquisition. (3) In the ALFFs-difference, some parietal lobe (SupraMarginal_L) was positively related to no-acquisition; there was no brain area negatively related to no-acquisition. (4) There was almost no common relevant brain areas among eyes-closed, eyes-open resting-states and their difference, except that left super occipital lobe was the negative relevant area in both eyes-closed and eyes-open resting-states.

Uncontrollable knowledge: (1) In the eyes-closed resting state, there was no brain area positively related to uncontrollable knowledge; some temporal lobe (Angular_L) was negatively related to uncontrollable knowledge. (2) In the eyes-open resting state, some frontal lobes (Precentral_R, Frontal_Mid_R, Frontal_Sup_Medial_R) were positively related to uncontrollable knowledge; there was no brain area negatively related to uncontrollable knowledge. (3) In the ALFFs-difference, some insula (Insula_R), hippocampus (Hippocampus_L, ParaHippocampal_L) and caudate (Caudate_L, Caudate_R) were positively related to uncontrollable knowledge; there was no brain area negatively related to uncontrollable knowledge. (4) There was no common relevant brain area among eyes-closed, eyes-open resting-states and their difference.

Half-controllable knowledge: (1) In the eyes-closed resting state, there was no brain area positively related to half-controllable knowledge; some fusiform (Fusiform_R) and temporal lobe (Temporal_Inf_L) were negatively related to half-controllable knowledge. (2) In the eyes-open resting state, there was no brain area positively related to half-controllable knowledge; some fusiform (Fusiform_R) and parietal lobe (Postcentral_R) were negatively related to half-controllable knowledge. (3) In the ALFFs-difference, there was no brain area positively related to half-controllable knowledge; some cerebelum (Cerebelum_4_5_L) was negatively related to half-controllable knowledge. (4) There was almost no common relevant brain areas among eyes-closed, eyes-open resting-states and their difference, except that right fusiform was the negative relevant area in both eyes-closed and eyes-open resting-states.

Controllable knowledge: (1) In the eyes-closed resting state, some fusiform (Fusiform_R) and parietal lobe (SupraMarginal_L) were positively related to controllable knowledge; there was no brain area negatively related to controllable knowledge. (2) In the eyes-open resting state, some frontal lobe (Frontal_Sup_L) and parietal lobe (Parietal_Inf_L, Parietal_Inf_R) were positively related to controllable knowledge; there was no brain area negatively related to controllable knowledge. (3) In the ALFFs-difference, there was no brain area positively related to controllable knowledge; some frontal lobe (Rolandic_Oper_L), hippocampus (Hippocampus_R), caudate (Caudate_L), cerebelum and vermis (Cerebelum_10_L, Vermis_9) were negatively related to controllable knowledge. (4) There was no common relevant brain area among eyes-closed, eyes-open resting-states and their difference.

Most relevant brain area of the four MET knowledge were different, except that right middle frontal gyrus was negatively related to no-acquisition, but positively related to uncontrollable knowledge; and that left caudate was positively related to uncontrollable knowledge, but negatively related to controllable knowledge; and that right fusiform was negatively related to half-controllable knowledge, but positively related to controllable knowledge.

## Discussion

### The behavioral and neuroimaging data were inconsistent with the PDP: The PDP did not establish

The PDP states that the correct inclusion-task response contains the incorrect exclusion-task response^5,8–9,26^; that is, they can be either positively (more likely), negatively (less likely) or not associated, and their relevant brain areas must in part overlap in neuroimaging data. The current study found that the two responses were negatively associated, which could be rather explained by, than prove the PDP. The ALFFs in the eyes-closed and eyes-open resting-state and their ALFFs-difference could predict the correct inclusion-task response and the incorrect exclusion-task response of the PDP. But brain areas related to the two responses were either completely different (for most brain areas) or opposite (for some brain areas), indicating that they were independent or competing. These neuroimaging results were inconsistent with the PDP. Therefore, the PDP did not establish.

### The behavioral and neuroimaging data were consistent with the MET: The MET established

The current study found that the two responses were negatively associated, and brain areas related to the two responses were either completely different (for most brain areas) or opposite (for some brain areas). These results were perfectly consistent with the MET, which considers that the correct inclusion-task response (C + A_1_) and the incorrect exclusion-task response (A_2_) are either independent or in opposition to each other, and they are likely to be either not correlated or negatively correlated (more likely), and the relevant brain areas of them should be either not overlap or in opposition to each other.

It is a pity that Zhang et al.^33^ did not make clear the similarities and differences between A_1_ and A_2_. The current study proposed that although both A_1_ and A_2_ can automatically act without consciousness, which is why they can be both called automatic responses, they are two different automatic responses or two different implicit knowledge that A_1_ can be controlled by task requirements, but A_2_ can not.

Therefore, the MET was used to categorize the 12 kinds of triplets in the SOC as delineating four MET knowledge, namely no-acquisition, uncontrollable knowledge, half-controllable knowledge, and controllable knowledge, whose controllability (degree of consciousness) was gradually increasing. Only half-controllable knowledge was in accordance with implicit knowledge as defined by the PDP, but it was present to a lesser extent than the other three knowledge types including uncontrollable knowledge whose incorrect exclusion-task response occurred more frequently than its correct inclusion-task response, which was contrary to the PDP. It is clear that either of the two responses were the mixture of four knowledge types, thus the MET used by the current study could get more precise knowledge than the PDP.

### ALFFs in eyes-closed and eyes-open resting-states and their difference were related to the four MET knowledge

The ALFFs in the eyes-closed and eyes-open resting-state and their ALFFs-difference could predict the four MET knowledge. The neuroimaging results were detailed analyzed in two opposite directions: (1) Because function of individual brain areas was detected by the previous literatures in implicit-sequence-learning consciousness, we still used it to simply explain the cognitive processes of the four MET knowledge (please see “Introduction” and the literatures below for details). (2) The cognitive processes manifesting themselves or being needed in behavioral characteristics of the four MET knowledge were used to speculate the function of relevant brain areas and the relationship among the different brain areas.

No-acquisition: (1) In the eyes-closed resting state, some frontal lobe was positively related to no-acquisition, which means the frontal lobe was adverse for knowledge acquisition, because no-acquisition was opposite to knowledge acquisition. The frontal lobe was responsible for consciousness^21,28,44^. Maybe the irrelevant, wrong and self-consciousness by frontal lobe hindered knowledge acquisition. Some occipital lobe was negatively related to no-acquisition, which means the occipital lobe was advantageous for knowledge acquisition. The occipital lobe was responsible for visual perception. (2) In the eyes-open resting state, some cerebelum and vermis were positively related to no-acquisition, which were responsible for motor process^9–10^. Some frontal lobes, occipital lobe and parietal lobe were negatively related to no-acquisition, of which frontal lobes and parietal lobe were responsible for consciousness^21,32^, and occipital lobe was responsible for visual perception. (3) In the ALFFs-difference, some parietal lobe was positively related to no-acquisition, which were responsible for consciousness. There was no brain area negatively related to no-acquisition. (4) There was almost no common relevant brain areas among eyes-closed, eyes-open resting-states and their difference, which means that they almost predicted no-acquisition (knowledge acquisition) diversely, except that left super occipital lobe was the negatively relevant area in both eyes-closed and eyes-open resting-states. (5) Because the no-acquisition has neither A_2_ nor C + A_1_, the advantageous brain areas can hinder either A_2_ or C + A_1_; the adverse brain areas can upgrade no-acquisition to uncontrollable knowledge by enhancing the A_2_, they can upgrade no-acquisition to half-controlled knowledge by enhancing both A_2_ and C + A_1_, and they can also upgrade no-acquisition to controlled knowledge by weakening A_2_ and enhancing C + A_1_. This requires specific analysis in conjunction with function of the brain areas. Summarily, some frontal lobe, cerebelum and vermis, parietal lobe was advantageous for no-acquisition (the opposite of knowledge acquisition). If some motor network of some participants was dominant relative to other participants or even other brain areas, it could make the participants press keys quickly and smoothly without the need to acquire sequence knowledg. If some consciousness network was dominant, it might produce much irrelevant, wrong and self-consciousness to interfere with the knowledge acquisition. However, some occipital lobes, frontal lobes and parietal lobe was adverse for no-acquisition. If the other consciousness network was dominant, it might produce much right consciousness to promote the knowledge acquisition. If some visual perception network was dominant, it might automatically obtain relationships between multiple locations to promote the knowledge acquisition. New measurement methods should be further developed to isolate the components of no-acquisition and then examine their relevant brain areas using both resting-state and task-state fMRI.

Uncontrollable knowledge: (1) In the eyes-closed resting state, there was no brain area positively related to uncontrollable knowledge. Some temporal lobe (Angular_L) was negatively related to uncontrollable knowledge, which was responsible for consciousness^32^. (2) In the eyes-open resting state, some frontal lobes were positively related to uncontrollable knowledge, which was responsible for consciousness. Maybe the irrelevant, wrong and self-consciousness by frontal lobe promoted uncontrollable knowledge. There was no brain area negatively related to uncontrollable knowledge. (3) In the ALFFs-difference, some insula, hippocampus and caudate were positively related to uncontrollable knowledge, of which insula and hippocampus were responsible for consciousness^13,20^, and caudate was responsible for implicit sequence learning^27^. Maybe the irrelevant, wrong and self-consciousness and the implicit sequence learning promoted uncontrollable knowledge. There was no brain area negatively related to uncontrollable knowledge. (4) There was no common relevant brain area among eyes-closed, eyes-open resting-states and their difference, which means that they predicted uncontrollable knowledge diversely. (5) Because the uncontrollable knowledge has only A_2_ but no C + A_1_, the advantageous brain areas can promote A_2_ but hinder C + A_1_; the adverse brain areas can downgrade uncontrollable knowledge to no-acquisition by weakening A_2_, they can upgrade uncontrollable knowledge to half-controlled knowledge by enhancing C + A_1_, and they can also upgrade uncontrollable knowledge to controlled knowledge by weakening A_2_ and enhancing C + A_1_. This requires specific analysis in conjunction with function of the brain areas. Summarily, some frontal lobe, insula, hippocampus and caudate was advantageous for uncontrollable knowledge. If some consciousness network was dominant, it might produce much irrelevant, wrong and self-consciousness to interfere with the knowledge controllability, because in the inclusion task, it would select the incorrect locations with their wrong consciousness, and in the exclusion task, it identified and avoided the false rule locations. If the implicit learning network was dominant, it might get much implicit and automatical knowledge to automatically select the rule locations in the exclusion task. However, some temporal lobe (Angular_L) was adverse for uncontrollable knowledge. If this consciousness network was dominant, it might produce much right consciousness to promote the knowledge controllability.

Half-controllable knowledge: (1) In the eyes-closed resting state, there was no brain area positively related to half-controllable knowledge. Some fusiform and temporal lobe were negatively related to half-controllable knowledge, which were responsible for consciousness^13,32^. (2) In the eyes-open resting state, there was no brain area positively related to half-controllable knowledge. Some fusiform and parietal lobe were negatively related to half-controllable knowledge, which were responsible for consciousness. (3) In the ALFFs-difference, there was no brain area positively related to half-controllable knowledge. Some cerebelum was negatively related to half-controllable knowledge, which was responsible for motor process. (4) There was almost no common relevant brain areas among eyes-closed, eyes-open resting-states and their difference, which means that they almost predicted half-controllable knowledge diversely, except that right fusiform was the negatively relevant area in both eyes-closed and eyes-open resting-states. (5) Because the half-controllable knowledge has both A_2_ and C + A_1_, these adverse brain areas can downgrade half-controllable knowledge to no-acquisition by weakening both A_2_ and C + A_1_; they can downgrade half-controllable knowledge to uncontrolled knowledge by enhancing A_2_ and weakening C + A_1_; they can also upgrade half-controllable knowledge to controllable knowledge by weakening A_2_ and enhancing C + A_1_. This requires specific analysis in conjunction with function of the brain areas. Summarily, some fusiform and temporal lobe, parietal lobe and cerebelum was adverse for half-controllable knowledge. If some consciousness network was dominant, on the one hand, it might produce much right consciousness to promote the knowledge completely controllability; on the other hand, it might produce much wrong consciousness to promote the knowledge completely uncontrollability or no-acquisition. If some motor network was dominant, on the one hand, it might produce much exercise control to promote the knowledge completely controllability; on the other hand, it might make participants press keys quickly and smoothly without consciousness to promote the knowledge completely uncontrollability or without the need to acquire sequence knowledge to promote no-acquisition. The specific mechanism needs further study. There was no brain area advantageous for half-controllable knowledge, which might be because some brain areas were in fact positively responsible for it, but there was no individual difference. Therefore the correlation analysis may wipe out the brain areas that play a fundamental role when there is no individual difference. The task-state fMRI need to explore the fundamental brain areas. Of course, the task-state fMRI may wipe out the brain areas that play a fundamental role for both the experimental trials and the control trials.

Controllable knowledge: (1) In the eyes-closed resting state, some fusiform and parietal lobe were positively related to controllable knowledge, which were responsible for consciousness. There was no brain area negatively related to controllable knowledge. (2) In the eyes-open resting state, some frontal lobe and parietal lobe were positively related to controllable knowledge, which were responsible for consciousness. There was no brain area negatively related to controllable knowledge. (3) In the ALFFs-difference, there was no brain area positively related to controllable knowledge. Some frontal lobe, hippocampus, caudate, cerebelum and vermis were negatively related to controllable knowledge, of which frontal lobe and hippocampus were responsible for consciousness, caudate was responsible for implicit sequence learning, and cerebelum and vermis was responsible for motor process. (4) There was no common relevant brain area among eyes-closed, eyes-open resting-states and their difference, which means that they predicted controllable knowledge diversely. (5) Because the controllable knowledge has no A_2_ but only C + A_1_, the advantageous brain areas can hinder A_2_ but promote C + A_1_; the adverse brain areas can downgrade controllable knowledge to no-acquisition by weakening both A_2_ and C + A_1_, they can downgrade controllable knowledge to uncontrolled knowledge by enhancing A_2_ and weakening C + A_1_, and they can also downgrade controllable knowledge to half-controlled knowledge by enhancing both A_2_ and C + A_1_. This requires specific analysis in conjunction with function of the brain areas. Summarily, some fusiform, parietal lobe and frontal lobe was advantageous for controllable knowledge. If some consciousness network was dominant, it might produce much right consciousness to promote the knowledge controllability. However, some frontal lobe, hippocampus, caudate, cerebelum and vermis was adverse for controllable knowledge. If the other consciousness network was dominant, it might produce much irrelevant, wrong and self-consciousness to interfere with the knowledge controllability or promote the knowledge half-controllability and no-acquisition; if some implicit learning network (caudate) was dominant, it might get much implicit and automatical knowledge to interfere with the knowledge controllability; if some motor network was dominant, it might make participants press keys quickly and smoothly without consciousness to promote the knowledge uncontrollability or without the need to acquire sequence knowledge to promote no-acquisition. The specific mechanism needs further study.

It can be seen from the above that the dominant/recessive brain areas may determine the amount and type of knowledge generated in implicit sequence learning, thus causing the consciousness preference among the participants, which could be one of the contents and sources of personality. In implicit learning, sensory participants were more likely to generate conscious knowledge, while intuitive participants were more likely to use intuition to complete tasks, so that they did not need to generate conscious knowledge^61^, Participants with high emotional openness were more likely to generate conscious knowledge than those with low emotional openness^6,62^. But these previous studies did not address individual differences in consciousness in terms of differences in brain function. The current study made such exploration and discovery.

Of course, with overall analysis, the four MET knowledge can be directly used as behavioral indicators to define cognitive processes in their relevant brain areas. Multiple brain areas coalesced into a whole to produce each MET knowledge. But detailed analysis above was necessary because each MET knowledge involves multiple cognitive processes, which can be separated at least by the two responses^33^. Overall analysis and detailed analysis do not contradict each other, but confirm each other.

There was almost no common relevant brain areas among eyes-closed, eyes-open resting-states and their difference for each of the four MET knowledge, which means that they almost predicted each of the four knowledge diversely, and it was necessary to set eyes-closed and eyes-open resting-states. Yan et al.^41^ found that the participants might have more non-specific or non-goal-directed visual information gathering and evaluation, and mind wandering or daydreaming during the eyes-open resting state than during the eyes-closed resting state. Nakano et al.^42,43^ found that in eyes-closed, subjects focused on internal feeling and self-consciousness, while in eyes-open, subjects turned to external stimulus processing, and the transition from eyes-closed to eyes-open was from internal feeling and self-consciousness to external stimulus processing. The current study found that some frontal lobes, parietal lobes, occipital lobes including fusiform, temporal lobes and cerebellum in the eyes-closed and eyes-open resting-state were related to the four MET knowledge, but there were more relevant brain areas in the eyes-open resting-state than in the eye-closed resting-state. The reason may be that in eyes-closed, subjects focused on internal feeling and self-consciousness, and brain activity in eyes-closed was only the baseline and the interference for the four knowledge in the external implicit sequence learning, so it would be less related to the four knowledge. Eyes-closed resting-state was suitable for find a global network for self-consciousness named the “rich club” in sleep and disturbed consciousness^44–47^, but was not as suitable as eyes-open resting-state for implicit-sequence-learning consciousness, which is the external stimulus consciousness with visual information gathering and evaluation. There was no study taking the ALFFs-difference that ALFFs in the eyes-open resting state minus ALFFs in the eyes-closed resting state as a quantitative transition sensitivity index from internal feeling to external stimulus or investigating the relationship between it and other cognition or behavior, although some literatures investigated its own psychological significance^41–43^. In the current study, we defined the ALFFs-difference as the quantitative transition sensitivity index from internal feeling to external stimulus, and found that the ALFFs-difference in some insula, hippocampus, caudate and cerebellum could predict the four MET knowledge, indicating more psychological significance. We can see that the relevant brain areas in the eyes-closed and eyes-open resting-state were the cerebral cortex responsible for vision, attention, cognitive control and consciousness, but the relevant brain areas in ALFFs-difference were the subcortical nucleus responsible for sensory awareness, memory and implicit sequence learning. We can call them the upper consciousness network and the lower consciousness network. Therefore it is necessary to set eyes-closed and eyes-open resting-states and use the ALFFs-difference as a quantitative transition sensitivity index to explore consciousness of external stimulus. Of course, they also apply to explore consciousness of internal feeling and self-consciousness.

All relevant brain areas of the four MET knowledge were different (for most areas) or opposite (for some areas such as right middle frontal gyrus, left caudate and right fusiform), that is, if some brain areas were related to one MET knowledge, they were not related to the other three MET knowledge, and if some brain areas were positively related to one MET knowledge, they were negatively related to other MET knowledge, which means that the four MET knowledge were independent or competitive from each other in relevant brain areas^33^. As the participants' control (degree of consciousness) was gradually increasing from no-acquisition to controllable knowledge, correspondingly, the brain areas in resting-state positively related to the four MET knowledge gradually changed from the consciousness network (some frontal lobe, parietal lobe) and the motor network (cerebelum and vermis), to the consciousness network (some frontal lobes, insula, hippocampus) and the implicit learning network (caudate), and then to the consciousness network (some fusiform, parietal lobe, frontal lobe and parietal lobe). The brain areas in resting-state negatively related to the four MET knowledge gradually changed from the consciousness network (some frontal lobe, parietal lobe) and visual perception network (some occipital lobes), to the consciousness network (some temporal lobe), then to the consciousness network (some fusiform, temporal lobe, parietal lobe) and the motor network (some cerebelum), and then to the consciousness network (some frontal lobe, hippocampus), the implicit learning network (caudate), and the motor network (cerebelum and vermis). Many relevant brain areas in resting-state were consistent with the results of the task-state researches^9–10,14,17,20,27,32^, which proved that the rationales of the resting-state were applicable to the current study of implicit-sequence-learning consciousness.

### The ecological validity of the MET was tested to be good

The results of the current study and Zhang et al. (2020) were compared: (1) Zhang et al. (2020) found that the correct inclusion-task response and the incorrect exclusion-task response were negatively associated in the SOA = 1350 ms group, *r* (21) = − 0.56, *p* < 0.01. The current study found that the two responses were negatively associated in the SOA = 1350 ms group too, *r* (65) = -0.58, *p* < 0.001. The correlation coefficients were almost the same. (2) Both Zhang, et al. (2020) and the current study found that in the SOA = 1350 ms group, the resting-state brain areas related to the two responses were either completely different (for most brain areas) or opposite (for some brain areas) between each other, although many of the relevant resting-state brain areas were different between the two studies. (3) Both Zhang et al. (2020) and the current study found that the relevant brain areas of the four MET knowledge were different or opposite between each other. (4) Zhang et al. (2020) found that corresponding with participants' control (degree of consciousness) gradually increasing from no-acquisition to controllable knowledge, the positively-relevant resting-state brain areas of the four MET knowledge types gradually changed from the sensory and motor network to the somatic sensorimotor network, and then to the implicit learning network, and then to the consciousness network. The negatively-relevant resting-state brain areas of the four MET knowledge gradually changed from the consciousness network to the sensory and motor network. The change trend was similar to the current study, but in the current study the consciousness networks played a important role in all four MET knowledge (see the last paragraph for details). The relevant brain areas were largely different between the two studies, which might be because they were different in the experiment settings and in the participants. The resting state itself may be changing, and the relationship between the resting state and implicit-sequence-learning consciousness may also be changing. To test these possibilities, in the future, resting state should be collected several times for the same group of participants, and then be correlated with the same implicit-sequence-learning consciousness. Overall, the results of the two studies were broadly consistent, but there were differences in detail.

There were problems with the ecological validity of the MET proposed by Zhang et al.^33^: (1) Participants completed implicit sequence learning while lying down and receiving task-state scanning. (2) Participants received 8 min post-task resting-state scanning before the inclusion/exclusion tasks. (3) Participants sat in front of a computer to do the inclusion/exclusion tasks, which was inconsistent with lying-down body posture in implicit sequence learning. (4) ALFFs in eyes-closed resting-state were related to the MET knowledge in implicit sequence learning, but participants did both the implicit sequence learning and the inclusion/exclusion tasks with eyes-open. These settings were different with the implicit sequence learning in previous studies and real life. Therefore, the current study made participants sit to do both the implicit sequence learning and the inclusion/exclusion tasks with the latter just after the former, and used eyes-closed and eyes-open resting-states and their difference to test the ecological validity of the MET. These settings were closer to implicit sequence learning in previous researches and real life. The behavioral and neuroimaging data were inconsistent with the PDP, but were still perfectly consistent with the MET, which indicates that the ecological validity of the MET is good. Although the current study and Zhang et al. (2020) were different in the experiment settings, the participants and the detail neuroimaging results, their behavioral and neuroimaging results were all perfectly consistent with the MET but inconsistent with the PDP, which indicates that the MET is a basic principle suitable for different experiment settings and participants. The ecological validity of the MET should be examined in more different settings in the future, such as more SOA, improbable sequences/transfer blocks, more body postures, movement, and so on. The MET can also be applied to the studies of implicit artificial grammar learning consciousness and perceptual consciousness to further test and expand its ecological validity.

## Conclusion

The current study made participants sit to do both the implicit sequence learning and the inclusion/exclusion tasks with the latter just after the former, and used eyes-closed and eyes-open resting-states fMRI and their difference to test the ecological validity of the MET in implicit sequence learning in the settings as previous studies and real life. The behavioral and neuroimaging data were perfectly consistent with the MET, which indicates that its ecological validity is good. ALFFs in eyes-closed and eyes-open resting-states and their difference were related to the four MET knowledge in implicit sequence learning diversely. The relevant brain areas of the four MET knowledge were different or opposite.
